# Exploring the mechanisms of resistance to *Teladorsagia circumcincta* infection in sheep through transcriptome analysis of abomasal mucosa and abomasal lymph nodes

**DOI:** 10.1186/s13567-018-0534-x

**Published:** 2018-04-27

**Authors:** Praveen K. Chitneedi, Aroa Suárez-Vega, María Martínez-Valladares, Juan José Arranz, Beatriz Gutiérrez-Gil

**Affiliations:** 1 Departamento de Producción Animal, Facultad de Veterinaria, Universidad de León, Campus de Vegazana s/n, 24071 León, Spain; 2 Departamento de Sanidad Animal, Facultad de Veterinaria, Universidad de León, Campus de Vegazana s/n, 24071 León, Spain; 3 Instituto de Ganadería de Montaña, CSIC-Universidad de León, 24346 Grulleros León, Spain

## Abstract

The present study exploited the RNA-seq technology to analyze the transcriptome of target tissues affected by the *Teladorsagia circumcincta* infection in two groups of adult ewes showing different statuses against gastrointestinal nematode (GIN) infection with the aim of identifying genes linked to GIN infection resistance in sheep. For this, based on the accumulated faecal egg count of 18 adult Churra ewes subjected to a first experimental infection with *T. circumcincta*, six ewes were classified as resistant and six others as susceptible to the infection. These 12 animals were dewormed and infected again. After humanitarian sacrifice of these 12 animals at day 7 post-infection, RNA samples were obtained from abomasal mucosa and lymph node tissues and RNA-Seq datasets were generated using an Illumina HiSeq 2000 sequencer. The distribution of the genes based on their expression level were very similar among the two different tissues and conditions. The differential expression analysis performed with two software (DESeq and EdgeR) only identified common differentially expressed genes (DEGs), a total of 106, in the lymph node samples which were considered as GIN-activated. The enrichment analysis performed for these GIN-activated genes identified some pathways related to cytokine-mediated immune response and the *PPARG* signaling pathway as well as disease terms related to inflammation and gastro-intestinal diseases as enriched. A systematic comparison with the results of previous studies confirmed the involvement of genes such as *ITLN2*, *CLAC1* and *galectins*, in the immune mechanism activated against *T. circumcincta* in resistant sheep.

**Electronic supplementary material:**

The online version of this article (10.1186/s13567-018-0534-x) contains supplementary material, which is available to authorized users.

## Introduction

In Spain, the dairy sheep production of indigenous sheep breeds is based on grazing livestock systems where gastrointestinal nematode (GIN) infections pose a major health problem to adult ewes and cause important economic losses [1]. Ovine resistance to the GIN infection is a highly complex character [1] and identification of genes influencing increased resistance to GIN infection would be of interest to enhance the efficiency of selection in commercial flocks through the use of molecular information. Hence, several studies have tried to identify QTL influencing indicator traits for GIN infection, such as fecal egg count (FEC), serum levels of IgA and pepsinogen. However, due to the major economic impact of GIN in young animals in countries such as the UK or Australia, most of these QTL studies are focused on lambs [2–5] whereas a limited number of studies have focused on adult sheep [6, 7] or combined data from aged ewes and lambs [1]. In a study carried out with adult sheep, Atlija et al. [7] suggested that some of the QTL identified that did not show overlapping with previous studies in lambs could be related to specific mechanisms of the immune response that is activated in adult animals. This would support the theory suggested by Stear et al. [8] that the genetic variation in FEC in lambs is predominantly a consequence of genetic variation in worm length and hence worm fecundity whereas, in contrast, mature sheep may be able to regulate not only fecundity, but also worm number.

As an alternative method to identify genes linked to GIN resistance, studies based on gene expression analysis can identify those genes whose expression may differ between animals and the gene pathways activated depending on the status against infection. In sheep infected by GIN, these kinds of studies based on expression analysis by RT-PCR of specific candidate genes [9–11], the use of microarrays [12, 13] or recently on the RNA-Seq technology [14, 15], have been mainly focused on young animals. For example, for lambs infected with GIN, different studies have shown that the animal status is dependent on the different type of host immune response that is activated against the nematodes in the abomasal (gastric) mucosa and abomasal lymph node [16, 17]. The *T. circumcincta* infection in lambs has been shown to trigger significant Th2 cytokine changes in mucosa with increased mucosa production of eosinophilia, mastocytosis and neutrophils. Upon GIN infection, the intelectins (*ITLN1*, *ITLN2* and *ITLN3*) [9, 10] and the interleukin (*IL*-*3*, *IL*-*4*, *IL*-*5* and *IL*-*13*) [18–20] transcripts have been consistently found to be upregulated to induce Th2 response in tissues like abomasal mucosa and lymph nodes of infected lambs whereas this expression was absent in naïve sheep. Irrespective of the breed and infective nematode species, some conserved gene expression responses were identified in relation to early inflammation in resistant lambs and in relation to a chronic inflammatory state in susceptible lambs [11].

In order to gain knowledge on the different gene expression responses activated in adult sheep between resistant and susceptible animals against *T. circumcincta* infection, the present study presents a comparative analysis of RNA-Seq datasets obtained from abomasal mucosa and abomasal lymph node obtained from two groups of adult Churra sheep previously classified as resistant and susceptible against this GIN infection. RNA-Seq has been shown as a powerful deep-sequencing technology that can help to elucidate previously inaccessible complexities underlying gene expression responses related to complex quantitative traits such as the resistance to nematode infection [21]. Hence, the study of complete target tissue transcriptomes presented here tries to provide a global picture of the different mechanisms activated as response to infection by GIN in adult sheep.

## Materials and methods

### Animals and experimental infections

Faecal samples of adult dairy ewes from four Churra dairy sheep flocks reared under semi-intensive management and belonging to the ANCHE breeders’ association (national association of Spanish Churra sheep breeders) were collected to initially assess the GIN infection levels after natural infection. Based on individual FEC, the farm showing the largest range for FEC was selected for additional sampling. In the selected flock, faecal samples were collected from a total of 119 adult ewes 3 months after the last deworming treatment. Based on the individual FEC measures, a total of 18 sheep (age range 6–8 years old) showing the most extreme FEC values were selected for our study. A first experimental infection (EI1) was performed based on a single oral administration of 50 000 *T. circumcincta* third stage larvae (L3) on these animals and after a treatment with one oral dose of ivermectin (0.2 mg/kg bw, Orame^®^, Merial, Spain). After this infection, collection of faeces was performed every 2 days, starting from day 14 to day 31 post-infection to calculate the accumulated FEC from each animal. Based on these values, six sheep were classified as susceptible and six sheep as resistant to infection by *T. circumcincta*. One month after the EI1, all these 12 selected ewes were treated with moxidectin by subcutaneous injection (0.2 mg/kg bw, Cydectin^®^, Zoetis, Spain) and 3 weeks later were exposed to a second experimental infection (EI2) with a single oral dose of 70 000 *T. circumcincta* L3. At day 7 after EI2, the animals were sacrificed by an intravenous injection with a lethal dose of 20 mL per sheep of sodium pentobarbital (Dolethal^®^, Vetoquinol, Spain). At necropsy, abomasal mucosa and lymph node samples for all animals were immediately collected in RNAlater™ Stabilization Reagent (Sigma-Aldrich, St. Louis, MO, USA). These samples were stored overnight at 4 °C and then frozen at −80 °C.

### RNA extraction, sequencing and bioinformatics analysis

mRNA were extracted from the abomasal mucosa and abomasal lymph node samples from the six resistant and six susceptible selected animals, using the Absolutely RNA miRNA Kit from Agilent (La Jolla, CA, USA). RNA integrity (RIN value) was analyzed using the agilent 2100 bioanalyzer (Agilent Technologies, Santa Clara, CA, USA). Considering both tissue samples the RIN values of the RNA samples ranged between 6.7 and 7.8. The preparation of the libraries and subsequent sequencing was performed with an Illumina HiSeq sequencer 2000, generating stranded paired-end reads of 75 bp with a depth of 30 million reads.

The read quality of each sample was assessed using FastQC V_0.11.5 software [22]. The high quality read samples were later aligned against the ovine reference genome (Oar_v3.1) using the alignment software STAR_v2.5.2b [23]. We used the option –*outSAMtype BAM Unsorted* to obtain an unsorted bam file instead of the sam file obtained by default with the STAR software. The unsorted aligned reads were indexed and sorted by read names using Samtools_v1.3.1 [24]. The sorted reads were counted for features (genes) in each sample using the HTSeq-count software [25] with the intersection-strict mode and reverse stranded option and using the information of the reference sheep genome annotation (Oar_v3.1.88.gtf) available at the FTP Download-Ensemble (release 88). This produces a feature list (gene list) with number of raw counts for each sample.

Before performing the differential gene expression analysis, we tried to quantify the abundance of all the annotated genes for each abomasal lymph node and abomasal mucosa sample analyzed. The gene expression levels were normalized by library size and gene length by calculating fragments per kilobase of exon per million fragments mapped (FPKM) with the RSEM software package [26] using the ensemble genome annotation (Oar_v3.1) as a reference. Initially the reference sheep genome annotation (Oar_v3.1.88.gtf) was preprocessed using the option *rsem*-*prepare*-*reference (*–*star)*. After that, we estimated the gene expression levels for each sample using the *rsem*-*calculate*-*expression (*–*star)* option of the RSEM program. The genes with at least 0.01 FPKM in each sample were considered as expressed and the mean gene expression across susceptible and resistant samples of each tissue were classified as high (> 500 FPKM), medium (10–500 FPKM) and low (< 10 FPKM) expressed genes based on their FPKM values.

The differential expression analysis was performed using two R based packages, EdgeR [27] and DESeq [28], with the raw counts from each sample obtained from HTSeq count. In these two software, the differential analysis of count data was performed in a similar way, but following different approaches to estimate normalization and dispersion. DESeq is, in comparison, less powerful but EdgeR is more sensitive to outliers [29]. Thus, after performing differential expression analysis between the resistant and the susceptible sample groups individually with the two software, only the genes commonly identified as differentially expressed genes (DEGs) by both EdgeR (FDR < 0.05) and DESeq (adjusted *P* < 0.05) were considered as “GIN-activated” DEGs. This double analysis is expected to reduce the presence of false positive results from our analysis. Based on log fold change (Log_2_FC) values, the DEGs were further classified as up-regulated in each group. These gene lists were subjected to three types of gene-set enrichment analysis [gene ontology (GO) analysis, KEGG pathway analysis and disease association analysis] using the web-based tool WebGestalt [30]. For these analyses, the human genome was considered as a reference and the parameters considered were the default statistical method Hypergeometric, multiple test adjustment with the BH method (Benjamini–Hochberg FDR), the significant level of adj*P *< 0.05. In addition, for a term to be considered significantly enriched a minimum of five genes were required for GO and disease association analyses, and a minimum of three genes were required for the KEGG pathway analysis.

## Results

### Infected sheep status confirmation

Based on the accumulated FEC estimation carried out on the 18 animals subjected to EI1, six animals were classified as “susceptible” (Chu6, Chur9, Chu11, Chu14, Chu17 and Chu19) and six as “resistant” (Chu1, Chu2, Chu7, Chu8, Chu15 and Chu21). Two of the individuals that were classified as “resistant” had zero counts throughout the control phase (Chu7, Chu15). The remaining five animals showed an uncertain profile between the two defined categories and therefore were discarded from further analysis. The mean accumulated FEC in the susceptible group was 5594 ± 2661 eggs per gram (epg) and 308 ± 338 epg in the resistant group.

### Gene expression level

**Figure 1:**
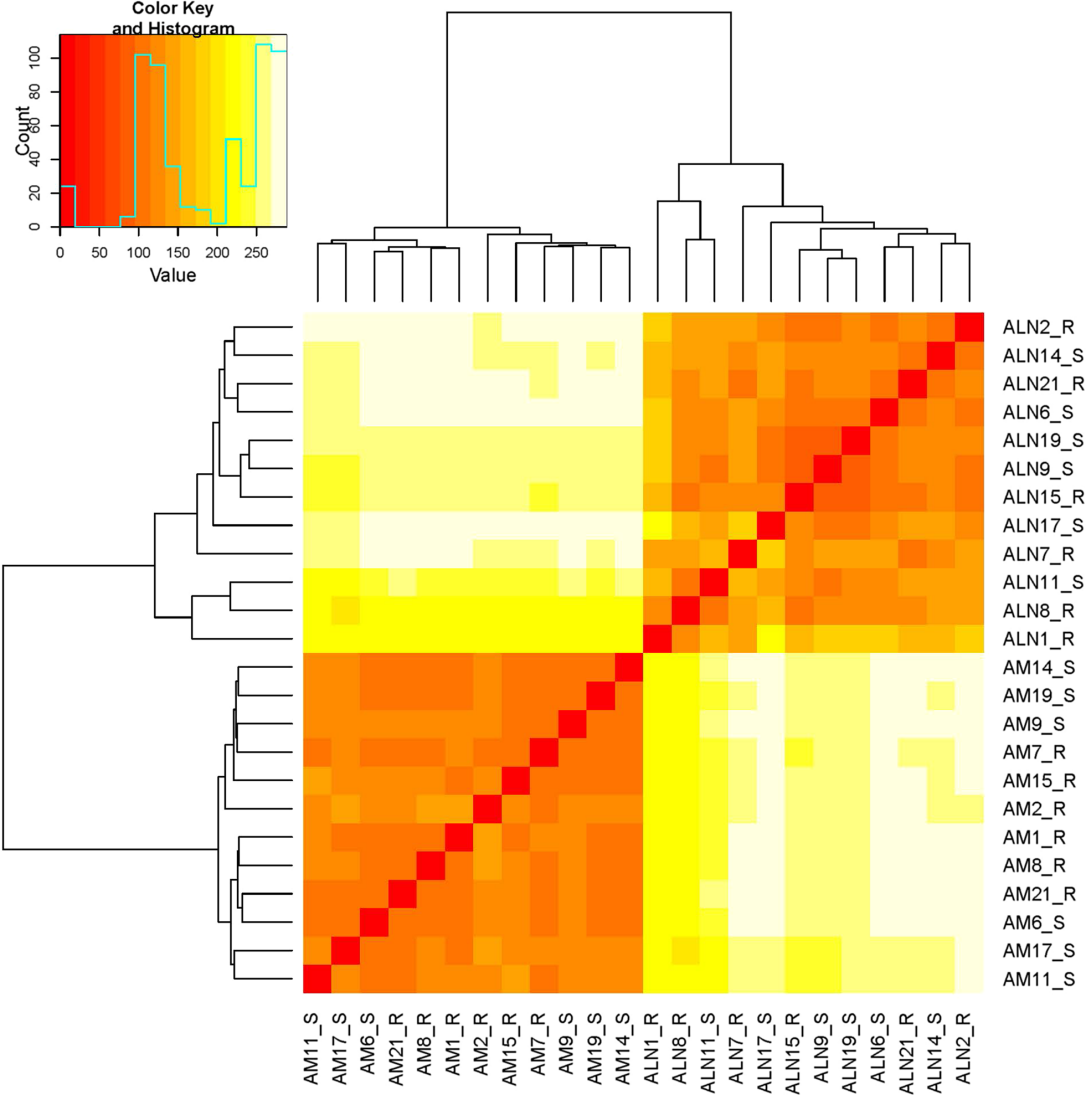
**Heatmap plot and hierarchical clustering of all the genes expressed in abomasal mucosa (AM) and abomasal lymph node (ALN) samples analyzed herein.** The plot includes the heatmap for samples of both tissues from 12 adult sheep subjected to an experimental infection with *T. circumcincta* and previously classified as resistant (“R”) and susceptible (“S”) based on a previous experimental infection with *T. circumcincta*.

A total of 24 mRNA samples, 12 from abomasal mucosa and 12 from abomasal lymph node, were considered for massive parallel sequencing. The FASTQC analysis showed that the quality of the sequenced reads for all the samples in two tissues under analysis were of high quality and thus, no trimming was performed. Across all the samples on average, around 72.3 and 79.2% of reads were uniquely mapped against the reference genome Oar_v3.1, for abomasal mucosa and abomasal lymph node tissues respectively. The aligned reads were sorted and counted against the list of annotated genes from the sheep reference genome Oar_v3.1. Out of the 27 054 annotated genes in this reference genome, on an average, around 16 210 (59%) genes in abomasal mucosa tissue sample data and 16 808 (62%) genes in abomasal lymph node tissue samples had at least one raw read count. The heatmap plot including the raw read count data of all abomasal mucosa and lymph node samples shows a clear distinction between both types of tissues (Figure 1). However, the total number of expressed genes was very similar among the two tissues and conditions, with an average of 15 627 ± 136 and 15 708 ± 154 genes expressed in abomasal mucosa and lymph node respectively (see Additional file 1). The distribution of the expressed genes across the different expression levels considered was also very similar in the two tissues and conditions (~1.31% of the genes classified as highly expressed genes; ~43% of the genes showing an intermediate expression level; ~55% of the genes with a low expression level). In the abomasal mucosa 179 and 180 genes were identified as highly expressed genes for the resistant and susceptible samples respectively. These highly expressed genes involved approximately 1.1% of the total FPKM in each group and were considered core genes (179 common core genes for the two compared groups, 0 specific core genes for the resistant group and 1 specific core gene for the susceptible group). In the lymph node 251 and 214 genes were identified as highly expressed genes for the resistant and susceptible samples, respectively (201 genes identified as core genes in the two conditions, 50 specific core genes of the resistant group and 13 specific core genes of the susceptible group). Considering the two tissues, we found 123 common genes in the highly expressed category (> 500 FPKM), 56 highly expressed genes specific to abomasal mucosa and 78 highly expressed genes specific to abomasal lymph node tissues (Additional file 2). By performing GO enrichment analysis with these common and specific highly expressed category genes, we found that the enriched terms resulting from the genes common to both tissues were related to basic physiology like *translation termination*, *translational elongation*, *mRNA catabolic process*, *RNA binding*, etc. (Additional file 3). The GO analysis for the core genes specific to abomasal mucosa highlighted terms such as *digestion*, *ATPase activity*, *hydrolase activity*, *ATP hydrolysis coupled proton transport* (Additional file 4), whereas in the analysis of the core genes specific to abomasal lymph node some of the enriched terms were *muscle contraction*, *collagen fibril organization*, *protein binding*, *extra cellular matrix*, etc. (Additional file 5). Within each tissue, the heatmap plot did not show a clear clustering of the samples based on the two contrasting groups, resistant versus susceptible samples. However, from the clustering obtained for the lymph node samples there were two resistant and two susceptible samples showing the most divergent expression pattern (Additional file 6).

**Figure 2:**
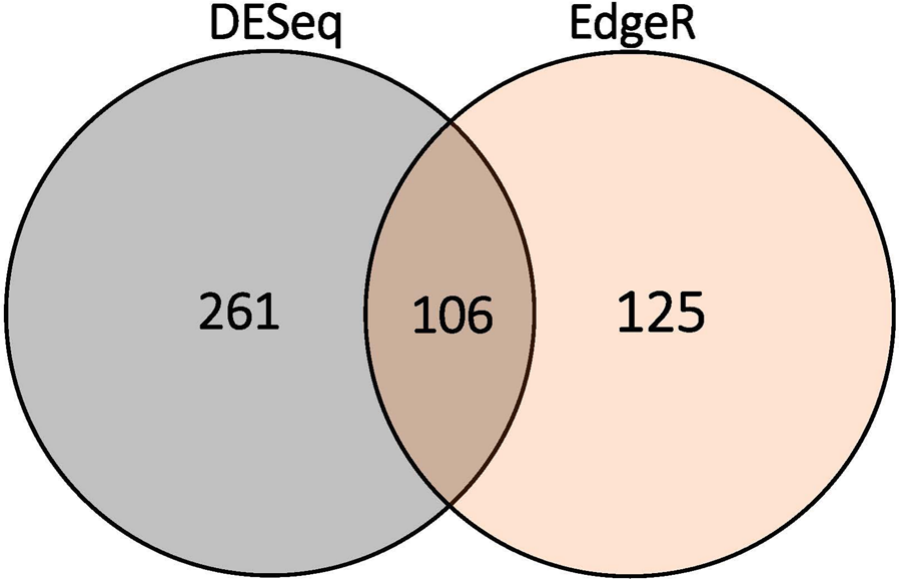
**Venn diagrams showing the total number of DEGs of abomasal lymph node samples with DESeq and EdgeR programs. ** The Venn diagram shows the total number of DEGs expressed with DESeq, EdgeR and the common DEGs between DESeq and EdgeR (GIN-activated genes) identified in the analysis of the 12 abomasal lymph node samples.

After performing the differential expression analysis with the abomasal mucosa RNA-Seq data, we found 33 DEGs (adjusted *P* < 0.05) using DESeq and no significant DEGs with EdgeR (FDR < 0.05) (Additional file 7). Out of these 33 significant genes, eight were up-regulated in resistant sheep and 25 were up-regulated in susceptible sheep. The differential expression analysis of the abomasal lymph node samples with DESeq (adjusted *P* < 0. 05) and EdgeR (FDR < 0.05) showed, respectively, a total of 261 and 125 DEGs. Among them, 106 genes were commonly identified as DEGs by both software and were considered as GIN-activated DEGs (Figure 2; Additional file 8). Out of these 106 common GIN-activated genes, 71 were up-regulated in resistant sheep and 35 were up-regulated in susceptible sheep. Note that for the intelectin gene, *ITLN*, a survey of orthologous genes indicates that this transcript corresponds to the bovine and human *ITLN2* gene.

The GO enrichment analysis performed for the GIN-activated DEGs with upregulated expression in resistant sheep shows nine significant terms in the *biological process* database, two of them related to cytokine response (*cytokine*-*mediated signaling pathway*, *cellular response to cytokine stimulus*), eight significant terms in the *cellular component* database and no significant term in the *molecular function* database (Additional file 9). On the contrary, for the GIN-activated DEGs showing upregulated expression in susceptible sheep, only three significant terms included in the *cellular component* database were identified (Additional file 9). The other two enrichment analyses performed, the KEGG pathway and disease association analyses, only identified significant terms for the GIN-activated DEGs showing upregulated expression in resistant sheep (Table 1). Among the three significant terms identified in the KEGG pathway analysis, we consider worth mentioning the *PPAR signaling pathway*, whereas the four significant terms identified in the disease association analysis were clearly related to the studied phenotype (*gastrointestinal neoplasms*, *intestinal/gastrointestinal diseases*, *inflammation*) (Table 1).

**Table 1:** **Results from enrichment analyses performed on the 71 GIN-activated DEGs up-regulated in resistant sheep identified for abomasal lymph node samples**

Database	Name	Nb. of genes	ID	Gene symbol	Statistics**
KEGG pathway analysis	Glycine, serine and threonine metabolism	3	260	*ALAS2*, *PSPH*, *GLYCTK*	C = 32; O = 3; E = 0.03; R = 103.67
					raw*P *= 3.33e−06; adj*P *= 9.99e−06
	PPAR signaling pathway	3	3320	*RXRG*, *PPARG*, *AQP7*	C = 70; O = 3; E = 0.06; R = 47.39;
					raw*P *= 3.59e−05; a dj*P *= 5.39e−05
	Metabolic pathways	7	1100	*CMBL*, *ALAS2*, *CKMT1A*, *PSPH*, *GLYCTK*,	C = 1130; O = 7; E = 1.02; R = 6.85;
				*ALPL*, *TST*	raw*P *= 6.15e−05; adj*P *= 6.15e−05
Disease association analysis	Gastrointestinal neoplasms	5	DB_ID:PA444257	*KRT20*, *PGC*, *B4GALNT2*, *PPARG*, *LGALS4*	C = 354; O = 5; E = 0.32; R = 15.62
					raw*P *= 1.66e−05; adj*P *= 0.0002
	Intestinal diseases	5	DB_ID:PA444632	*KRT20*, *PGC*, *PPARG*, *SLC22A4*, *LGALS4*	C = 331; O = 5; E = 0.30; R = 16.70
					raw*P *= 1.20e−05; adjP = 0.0002
	Inflammation	5	DB_ID:PA444620	*SFTPD*, *PPARG*, *PF4*, *CLCA1*, *IL5RA*	C = 435; O = 5; E = 0.39; R = 12.71
					raw*P *= 4.43e−05; adj*P *= 0.0003
	Gastrointestinal diseases	5	DB_ID:PA444256	*KRT20*, *PGC*, *PPARG*, *SLC22A4*, *LGALS4*	C = 413; O = 5; E = 0.37; R = 13.39
					rawP = 3.46e−05; adj*P *= 0.0003

**C: the number of reference genes in the category, O: the number of genes in the gene set and also in the category, E: the expected number in the category, R: ratio of enrichment, rawP: *P* value from hypergeometric test, adjP: *P* value adjusted by the multiple test adjustment.

## Discussion

The mechanisms of sheep resistance to GIN infections involve complex immune responses. In relation to the specific infection with *T. circumcincta*, several studies have previously been carried out in lambs showing that, as a local immune response to *T. circumcincta* infection, the levels of IgA and eosinophils were increased in the abomasal tissues of infected lambs [31–34]. Based on cDNA microarray-based studies, several up-regulated transcripts were found in abomasal tissues of *T. circumcincta* infected lambs and confirmed the activation of the Th2-type immune response in mucosa tissue, including eosinophilia and mastocytosis [35–37]. In this study, we tried to decipher the immune mechanisms activated in adult sheep during infection with *T. circumcincta* through the transcriptome analysis of the two main tissues targeted by the infection, the abomasal mucosa and the abomasal lymph node.

Although our initial purpose was to classify the animals to be studied as resistant and susceptible based on natural infection, the lack of homogeneity regarding the infection level when working with animals in pasture determined the need to perform a first experimental infection (EI1) to ensure similar infection levels among all the animals included in the study.

The results of the differential expression analyses performed in the two tissues studied suggest that at day 7 post-infection there is not a clear differential gene expression response in the abomasal mucosa (due to the lack of DEGs identified by EdgeR). However, a differential response in the abomasal lymph node was clearly observed, based on the identification of 106 genes commonly identified as DEGs by the two software. We acknowledge that the results from the differential expression analyses performed with EdgeR and DESeq may include a similar fraction of false negative, as they both rely on a negative binomial model and use the false discovery rate procedure [38] to adjust for multiple testing. In this regard, Zhang et al. [39] suggested that taking the intersection of DEGs from two or more tools is recommended if the number of false positives is a major concern in the study. Following this, we considered for further analyses those genes that were identified as DEGs by the two implemented methods, reducing the initial number of DEGs from 261 and 125, respectively for DESeq and EdgeR, to 106 genes defined as GIN-activated DEGs.

Hence, focusing on the 106 GIN-activated DEGs identified in the complete analysis of the lymph node samples, some of the significant terms identified by the enrichment analyses showed a clear correspondence with immune response mechanisms. In particular, the two GO terms related to cytokines highlighted by the GO analysis of genes upregulated in resistant ewes (*cytokine*-*mediated signaling pathway* and *cellular response to cytokine stimulus*) were related to the same five genes (*PALM3*, *DUOX2*, *PPARG*, *PF4*, *IL5RA*) (Table 1). The role of *PPARG*, *DUOX2* and *IL5RA* genes in relation to the immune response has been previously reported in different nematode infection studies [11, 20, 40]. In our study, the *PPARG* gene also supported the identification of the *PPAR* signaling pathway in the KEGG analysis, together with the *RXRG* and *AQP7* genes. The *PPARG* gene was also linked to many of the significant related terms highlighted by the disease association analysis (*gastrointestinal neoplasms*, *intestinal diseases*, *inflammation* and *gastrointestinal diseases*, Table 1). The *IL5RA* gene also supported the identification of the term *inflammation* as enriched in the disease association analysis (Table 1).

Some of the DEGs identified in our study have been previously identified as DEGs by other sheep gene expression analysis in relation to gastrointestinal nematode infection. To help assess the level of correspondence of our results with other studies, we summarize the results of our comparative literature survey in Table 2. We show that 10 out of the 106 GIN-activated genes identified in our study have been previously reported to show a modified expression due to GIN infection: *ITLN*, *LYZ*, *LOC443162* (*galectin 14*), *LGALS4* (*galectin 4*), *CLCA1*, *ALPL*, *PDZK1IP1*, *PPARG*, *KRT5*, *IL5RA* (genes highlighted in bold font in Table 2). Note that for the gene annotated as *ITLN* in the sheep genome, the ortholog analysis clearly shows that it corresponds to the *ITLN2* gene, which has been identified by other studies as activated by the GIN infection. The rest of genes presented in Table 2 not highlighted in bold font are genes that belong to the same family as some of the GIN-activated genes reported here.

**Table 2:** **Gastro-intestinal nematode infection studies that have overlapping genes with our study**

Animal age/breed	Infection status	Tissue used for study	*Nematode species*	*Genes* ^a^	Technique	Study
Yearling Scottish Blackface sheep	Challenged naive sheep	Abomasal mucosa	*T. circumcincta*	***ITLN2***, ***IL4***, ***galectin 14***	RT-PCR, Western blot	[9]
Yearling sheep	Immune day 5 vs naïve day 5	Abomasal mucosa	*T. circumcincta*	***CLCA1***, ***PDZK1IP1***	cDNA microarray, RT-PCR, QT-PCR	[37]
Yearling sheep	Naive sheep	Abomasal mucosa	*T. circumcincta*	***ITLN2***, *ITLN1*, *ITLN3* ***galectin 4***, *galectin 1*	cDNA microarray, RT-PCR, QT-PCR	[37]
Yearling sheep	Naive day 5 vs naive day 0	Abomasal mucosa	*T. circumcincta*	***LYZ***, *MMP13*	cDNA microarray, RT-PCR, QT-PCR	[37]
6 months old Merino-cross wethers	Infected sheep	Abomasal mucosa	*H. contortus*	***ITLN2***, ***CLCA1***, *interleukins*	Sequential microarray (across all arrays)	[12]
6 months old Merino-cross wethers	Infected sheep	Abomasal mucosa	*H. contortus*	***ALPL***, ***PDZK1IP1***	Sequential microarray (day 22 vs day 3 biopsies)	[12]
Lambs	Primary challenge vs Tertiary challenge	Lymph node	*T. colubriformis*	***PPARG***, ***KRT5***, *SLC31A2*, *KRT18*	micro array data, QT PCR	[13]
Lambs	Resistant sheep	Abomasal mucosa	*T. colubriformis*, *H. contortus*	*DUOX1*, *IL2RA*, *IL10*	RT-PCR	[11]
Adult sheep	Immune sheep	Abomasal mucosa	*T. circumcincta*	***LYZ***, ***ITLN2***, *ITLN3*, *CLCA*, *KRT10*, *KRT8*, *KRT19*	SDS-PAGE and Shortgun proteomics	[44]
Lambs Scottish Blackface	Susceptible sheep	Lymph node	*T. circumcincta*	*SLC30A2*, ***galectin 14***	RNA-seq	[14]
1 year old canaria hair breed (CHB) and canaria sheep (CS)	Infected CHB	Abomasal mucosa	*H. contortus*	*galectin 15*, *IL5*, ***ALPL***, *MMP1*, *MMP11*, *MMP14*, *MMP2*	RNA-seq, RT-PCR	[15]
1 year old canaria hair breed (CHB) and canaria sheep (CS)	Infected CHB and CS	Abomasal mucosa	*H. contortus*	*SLC2A3*, *IL1RL1*	RNA-seq, RT-PCR	[15]
Lambs Scottish Blackface	Resistant vs Control	Lymph node	*T. circumcincta*	***IL5RA***, *IL13*, *IL13RA2*, *IL1RL1*, *IL4*, *SLC9A4*	cDNA microarray, RT-PCR	[20]
Lambs (Scottish Blackface x Leicester)	Infected sheep	Abomasum, lymph node	*T. circumcincta*	***ITLN2***, *ITLN1*, *ITLN3*	Semi-quantitative RT-PCR	[10]

^a^List of genes whose expression level is affected by GIN-infection. Those highlighted in bold font were also identified as GIN-activated differentially expressed genes in our study.

Among the list of ten genes commonly identified by our study and other authors to be responsive to GIN infection, *PPARG*, *LYZ*, and *IL5RA* are directly related to inflammatory response. *PPARG* encodes for the *peroxisome proliferator activated receptor gamma*, which is a ligand activated transcription factor that regulates adipocyte differentiation and glucose homeostasis, but it has also been recognized as playing a key role in the immune response through its ability to inhibit the expression of inflammatory cytokines and to direct the differentiation of immune cells towards anti-inflammatory phenotypes [41]. In our study the *PPARG* gene was found to be upregulated in resistant ewes compared with susceptible ewes. A modified expression pattern of the *PPARG* gene in relation to the infection by *T. colubriformis* and *H. contortus* in sheep has already been reported by Andronicos et al. [13] (Table 2). A study carried out on naïve Perendale lambs suggested this gene plays a role in coordinately regulating genes more highly expressed in the intestine of the susceptible lambs [35]. In mice, high expression of the *PPARG* gene was found in response to nematode infection and the mice lacking the *PPARG* gene were unable to mount protective immune response to nematode infection. Hence, *PPARG* was suggested as a factor that drives type 2 responses in worm infection [40].

The interleukin gene *IL5RA* was also a GIN-activated gene up-regulated in resistant sheep. This gene supported the enriched GO terms *cytokine*-*mediated signaling pathway* and *cellular response to cytokine stimulus* and the disease association related term *Inflammation*. This gene was also found to have an increased expression in resistant Scottish Blackface lambs to *T. circumcincta* by Gossner et al. [20] (Table 2). IL5RA is required for the biological activities of IL5 to promote eosinophil-mediated activation and recruitment into tissues in acute inflammatory responses [42]. Increased levels of eosinophils have been classically linked to resistance to *Trichostrongyle* parasites in sheep. Hence, eosinophilia has been suggested as a marker of resistance to *T. circumcincta* in Scottish Blackface lambs [34], whereas a more pronounced eosinophilia has been documented in animals bred for increased resistance to *T. colubriformis* infection [43].

The *LYZ* gene encodes lysozyme, a protein with antibacterial activity. In our study this gene was the most highly up-regulated GIN-activated gene in susceptible sheep. This observation agrees with the work of Knight et al. [37] who reported, in the abomasal mucosa, down-regulation of members of the gastric lysozyme family (*LYZ 1A*, *2A*, *3A* and *4A*) in immune versus naïve sheep at days 2 and 5 post-challenge with *T. circumcincta* (Table 2). Gastric lysozyme genes are highly expressed in the ovine abomasum and are thought to act as a major digestive enzyme of the peptidoglycan cell walls of bacteria entering from the rumen, functioning at low pH. Other studies, however, have found genes of the lysozyme family to be up-regulated in abomasal epithelial extracts from previously infected sheep versus naïve sheep [37, 44] (Table 2). Also the alterations in lysozyme production have been suggested to contribute to the resulting nutritional loss seen in infected animals [37].

Another GIN-activated gene up-regulated in resistant sheep based on our study, *CLCA1*, has also been identified as up-regulated in immune lambs in the study reported by Knight et al. [37] and in the *H. contortus* challenged yearling lambs analyzed by Rowe et al. [12]. The encoded protein of this gene is thought to act as a multifunctional signaling protein, including an early modulator of immune responses by regulation of cytokines [45]. Proteins of the CLCA family may contribute to parasite expulsion by being responsible for mucus hydration across the gut epithelium and smooth muscle contraction [12] (Table 2).

Another transcript related to mucous cells, *ITLN*, was the third most highly up-regulated in resistant sheep in our study. The intelectin 1 and 2 are protein coding genes related to carbohydrate binging. Other studies have already reported an increased expression of this gene in relation to the infection response of lambs to *T. circumcincta* [9, 37] and *H. contortus* infection [12]. The early expression of *ITLN* post-challenge in immune yearling sheep compared with naïve yearling sheep was suggested as a protective role and it may also alter the characteristics of mucus leading to worm entrapment [9] (Table 2). Up-regulation of *ITLN* has also been reported in resistant mice in response to *Trichuris muris* infection [46]. The expression of *ITLN1* and *ITLN3* was found in lymph node tissues in response to *T. circumcincta* infection in Scottish Blackface x Leicester lambs but no protein expression was found on immunohistochemistry [10] (Table 2). In cattle, the expression of *ITLN2* was reported in abomasal mucosa tissue of resistant 12 month old Angus cattle [47]. In murine models the intelectin transcript plays a key role in the expression of *IL*-*25*, *IL23* and has been found to amplify type 2 immune response in asthma and atrophic dermatitis conditions in humans [48]. Overall, our study supports the role of some genes such as *ITLN2*, *CLCA1* in adult sheep GIN resistance. These two genes have been previously reported to be increased in immune lambs and to be up-regulated in resistant adult sheep. Whereas the expression of these genes in lambs was found to be affected by infection in abomasal mucosa, our study did not identify an altered pattern in that tissue but only in the lymph node.

Three genes identified in our study as GIN-activated, and up-regulated in resistant sheep, belong to the galectin family, *LOC443162* (Gal-14), *LGALS4* (Gal-4) and *LOC101102156* (Gal-9). Galectins mediate innate and adaptive immune functions by modulating the activity of complement receptor 3, macrophage and dendrocyte adhesion to lymphocytes [49]. The expression of *Gal*-*14* was found in adult sheep after exposure to an allergen (house dust mites) and may be responsible for eosinophil function and inflammation due to allergy [50]. There was a significant up-regulation of *Gal*-*4* in challenged naïve yearling lambs after infection with *T. circumcincta* L3 [37]. The expression of *Gal*-*14* was maximum at day 10 post-challenge in yearling Scottish Blackface lambs previously infected with *T. circumcincta* [9]. In Scottish Blackface lambs, the differential expression of the *Gal*-*14* gene was reported in abomasal lymph node of resistant animals after 14-day post-infection with *T. circumcincta* [14] (Table 2). In addition, a role of galectins has been widely reported in reference to different host parasite interactions and these proteins appear to be responsible for adhesion of pathogens to host cells and host adaptive immunity [51].

Our list of GIN-activated genes also includes some genes belonging to the same gene families reported in other relevant GIN studies. Some of these include genes from the matrix metallopeptidase (MMP) family (*MMP28*) and solute carrier family genes (*SLC22A4* and *SLC25A34*, *SLC38A2*). The role of these genes in inflammatory diseases has been previously described. Hence, different matrix metallopeptidase family genes were related to inflammatory response in humans, in particular the *MMP28* gene was responsible for altering inflammatory response in mice [52]. Also, the expression of other SLC family members were reported in response to nematode infection in both susceptible and resistant sheep and variants in the *SLC22A4* gene have been associated to Inflammatory Bowel disease in humans [53]. Whereas, high expression of *SLC30A2* in susceptible sheep in response to *T. circumcincta* infection has been reported in Scottish Blackface lambs [14], *H. contortus* infection shows a significant impact on the expression of the *SLC2A3* gene in Canary sheep breeds [15] (Table 2).

It is noteworthy that there is a list of about 86 genes identified as GIN-activated in our study that have not been reported or that do not belong to gene families considered as responsive to GIN infection in previous studies (Additional file 10). Interestingly, for some of these genes we found connection with the immune response (*PGC*, *SFTPD*, *TUBA4A*, *SST*, *BPIFB1*, *PF4*, *B4GALNT2*, *JCHAIN*, *AQP7*, *KLHL25*, *NEDD4*, *ANO6*). Because most of the previously reported studies considered in our comparative survey (Table 2) are focused on lambs, we think that the genes reported here in Additional file 10 might indicate genes that are specifically activated in adult sheep and not in lambs.

Other genes identified as DEGs by other studies have not been found as GIN-activated genes in the present work. Hence, genes belonging to the chemokine (*CCL*, *CXCL*) and the collagen families (*COL9A2*, *COL6A5*), or the interferon gamma gene (*IFNG*), etc. which are reported as DEGs in abomasal lymph node in resistant lambs as a response to *T. circumcincta* infection [14, 20] were not found as DEGs in our study. This may be due to differential activation of the immune response to *T. circumcincta* infection in adult sheep compared with young animals although some similar pathways were activated. Also the differences among the different studies regarding the species responsible for gastrointestinal parasite infection, the different experimental approaches (natural vs artificial), the post-infection sample extraction timings, or the environmental conditions (dry vs humid climates) are other major factors that could explain discrepancies related to the specific genes activated during GIN infection.

Also some of these genes may have been identified as DEGs in our study by one of the two DE analyses performed. Some genes such as *CENPN*, *FABP4*, *HSH2D*, *KIF2C*, *KIFC1*, *MMP1*, *NDC80*, *NEK2*, *PKMYT1*, *SFN*, *SPAG5*, *UBE2C*, *UHRF1*, which have been reported as GIN related by other studies were only identified as DEGs by DESeq but not by EdgeR. Hence, the approach implemented in our paper to avoid false positives, may have determined a restrictive threshold and we may have lost some important genes in our final list of GIN-activated genes.

Our study provides a global picture of the changes that occur in the transcriptome of target tissues, abomasal mucosa and abomasal lymph node, as a response to *T. circumcincta* infection in resistant and susceptible adult sheep. Whereas at day 7 post-infection, we did not find a differential response between the two compared groups in abomasal mucosa, a total of 106 genes were identified to show a distinct pattern between the two contrasted groups in the lymph node samples. The comparative study of our results with the available literature has shown remarkable coincidences for some of these genes with other gene expression studies related to GIN infection in lambs. Hence, the expression of genes such as *ITLN2*, *CLCA1*, *galectin 14*, etc. appear to be consistently affected by nematode infection, in both lambs and adult sheep. The differential expression of some immune-related genes reported in the present study as a response to *T. circumcincta* and that are not coincident with previous studies focused on lamb animals could indicate immune mechanisms that are specifically activated in adult animals (e.g. *PGC*, *SFTPD*, *TUBA4A*, *SST*, *BPIFB1*, *PF4*, *B4GALNT2*, *JCHAIN*, *AQP7*, *KLHL25*, *NEDD4*, *ANO6*). The RNA-Seq technology has shown here to be an appropriate platform to investigate the molecular mechanisms underlying the immune response to nematode infection in adult sheep. Future studies will focus on the genetic variability of the GIN-activated genes reported here with the aim of identifying potential candidate mutations that could be directly implemented in selection programs to increase GIN resistance in commercial sheep populations.

## Additional files

**Additional file 1.** **Gene expression levels in the two tissues and conditions studied.** Distribution of gene expression levels for the genes expressed in the transcriptome of abomasal mucosa and abomasal lymph node samples for the two groups of animals compared in the present study.

**Additional file 2.** **Venn diagrams showing the number of genes identified highly expressed genes (> 500 FPKM) by the analysis of the abomasal mucosa and abomasal lymph node transcriptomes.** The gene expression levels were normalized by library size and gene length by calculating Fragments Per Kilobase of Exon Per Million Fragments Mapped (FPKM). A total number of 123 genes were identified as highly expressed in the two tissues, whereas 56 and 78 genes were highly expressed specifically in the abomasal mucosa and the abomasal lymph node samples respectively.

**Additional file 3.** **Gene-set enrichment analysis (GO) for the highly expressed genes in both tissues studied.** Significant terms from the Gene Ontology (GO) enrichment analysis performed for the genes identified as highly expressed genes (≥ 500 FPKM) in both tissues studied, abomasal mucosa and abomasal lymph node tissue.

**Additional file 4.** **Gene-set enrichment analysis (GO) for the highly expressed genes in abomasal mucosa tissue.** Significant terms from the Gene Ontology (GO) enrichment analysis performed with WebGestalt for the genes identified as highly expressed (≥ 500 FPKM) specifically in abomasal mucosa.

**Additional file 5.** **Gene-set enrichment analysis (GO) for the highly expressed genes in lymph node tissue.** Significant terms from the Gene Ontology (GO) enrichment analysis performed with WebGestalt for the genes identified as highly expressed (≥ 500 FPKM) specifically in lymph node.

**Additional file 6.** **Heatmap plot of the lymph node transcriptome of six resistant and six susceptible adult ewes based on raw read counts.** The heatmap plot of raw RNA read counts did not show a clear differentiation between the two groups of samples (Resistant vs Susceptible). But the most divergent clusters of two resistant (ALN7_R and ALN1_R) and two susceptible (ALN19_S and ALN9_S) samples can be observed.

**Additional file 7.** **List of genes identified as significantly differentially expressed genes (DEGs) by the DESeq analysis for the abomasal mucosa samples.** The log2FoldChange value obtained from the DESeq analysis is given for the genes identified as up-regulated in the resistant group and for the genes identified as up-regulated in the susceptible group.

**Additional file 8.** **List of DEGs identified as GIN-activated genes for the abomasal lymph node samples (Resistant and Susceptible ewes to** ***T. circumcincta*** **infection).** The log2FoldChange value obtained from the EdgeR and DESeq analyses are given for the genes identified as GIN-activated in the resistant group and for the genes identified as GIN-activated in the susceptible group.

**Additional file 9.Significant terms from the Gene Ontology (GO) enrichment analysis performed with WebGestalt for the genes identified as GIN-activated in abomasal lymph node tissue.** The results of the GO enrichment analysis are provided separately for the genes identified as up-regulated in the resistant and susceptible groups.

**Additional file 10.** **Novel candidate genes for GIN resistance response presented in this work.** List of DEGs identified as GIN-activated genes in the analysis of the abomasal lymph node samples that have not been previously reported as GIN related genes and that could be related to the immune mechanisms specifically activated in adult sheep. The log2FoldChange obtained in the analyses performed with EdgeR and DESeq are provided for each gene.
