# Supplementary figures and images for: Exploring the mechanisms of resistance to Teladorsagia circumcincta infection in sheep through transcriptome analysis of abomasal mucosa and abomasal lymph nodes
Source: Vet Res. 2018 Apr 27;49:39. doi: 10.1186/s13567-018-0534-x (PMC5922024; doi:10.1186/s13567-018-0534-x)

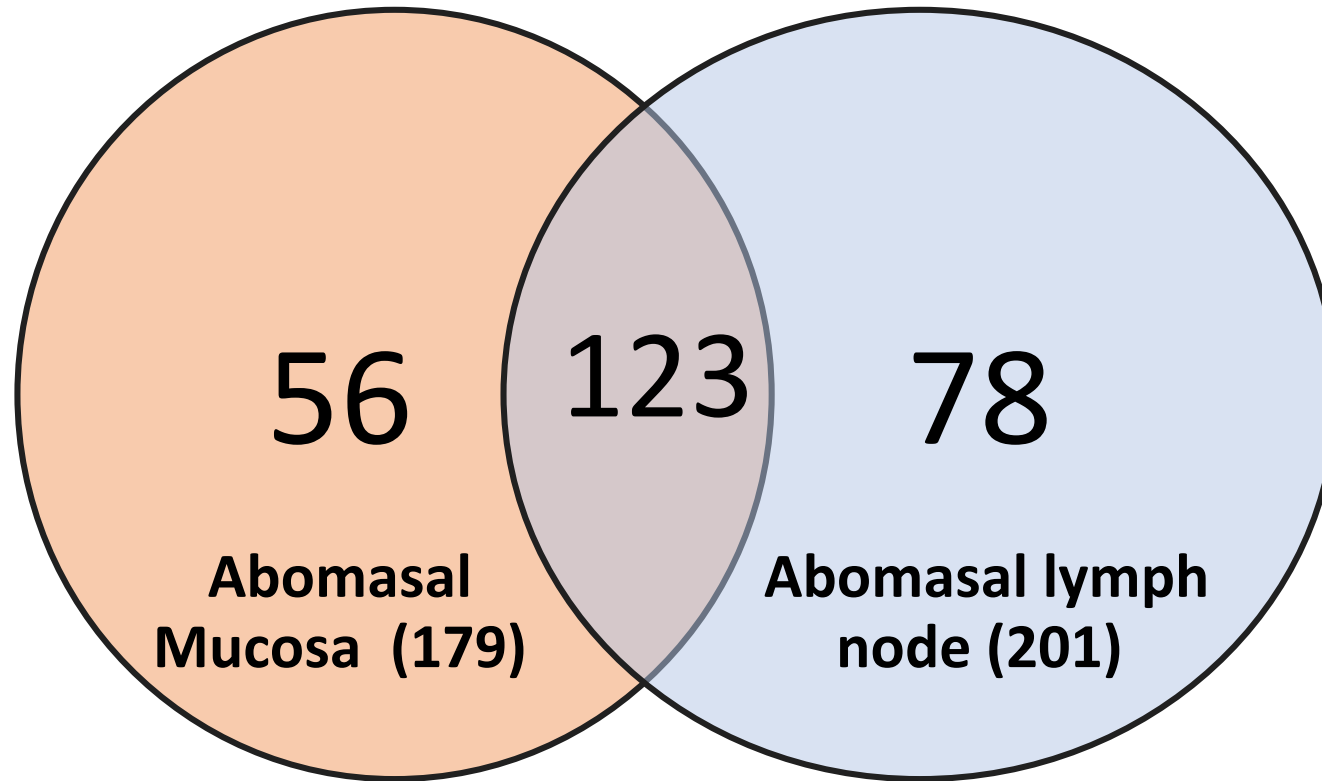

Supplement: Supplementary file 2 — Additional file 2. Venn diagrams showing the number of genes identified highly expressed genes (> 500 FPKM) by the analysis of the abomasal mucosa and abomasal lymph node transcriptomes. The gene expression levels were normalized by library size and gene length by calculating Fragments Per Kilobase of Exon Per Million Fragments Mapped (FPKM). A total number of 123 genes were identified as highly expressed in the two tissues, whereas 56 and 78 genes were highly expressed specifically in the abomasal mucosa and the abomasal lymph node samples respectively. [file 13567_2018_534_MOESM2_ESM.pdf]
