# Supplementary material for: Exploring the mechanisms of resistance to Teladorsagia circumcincta infection in sheep through transcriptome analysis of abomasal mucosa and abomasal lymph nodes
Source: Vet Res. 2018 Apr 27;49:39. doi: 10.1186/s13567-018-0534-x (PMC5922024; doi:10.1186/s13567-018-0534-x)
Supplement: Supplementary file 6 — Additional file 6. Heatmap plot of the lymph node transcriptome of six resistant and six susceptible adult ewes based on raw read counts. The heatmap plot of raw RNA read counts did not show a clear differentiation between the two groups of samples (Resistant vs Susceptible). But the most divergent clusters of two resistant (ALN7_R and ALN1_R) and two susceptible (ALN19_S and ALN9_S) samples can be observed. [file 13567_2018_534_MOESM6_ESM.pdf]

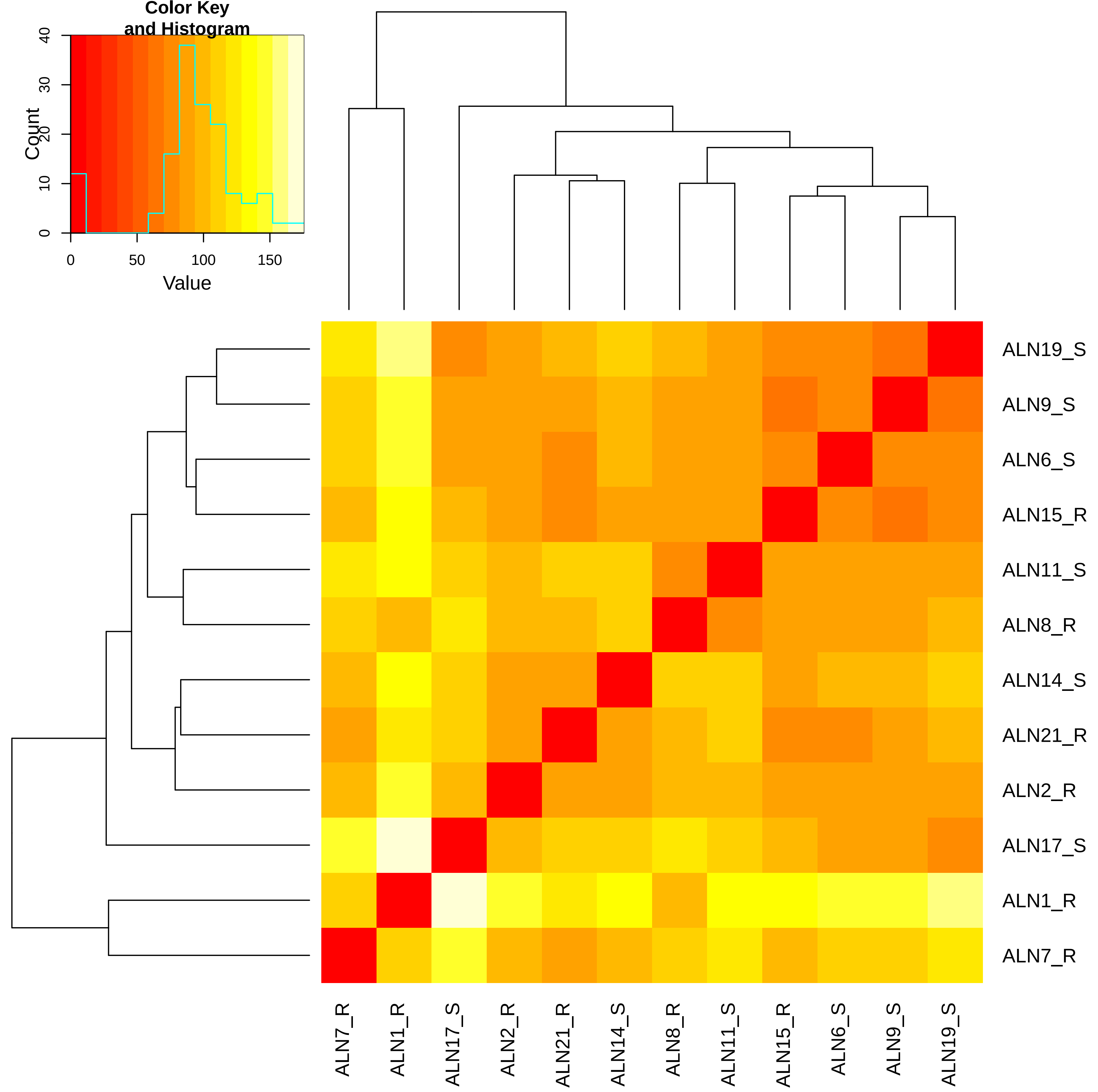

Color Key
and Histogram
Count
0
10
20
30
40
0
50
100
150
Value
ALN19_S
ALN9_S
ALN6_S
ALN15_R
ALN11_S
ALN8_R
ALN14_S
ALN21_R
ALN2_R
ALN17_S
ALN1_R
ALN7_R
ALN7_R
ALN1_R
ALN17_S
ALN2_R
ALN21_R
ALN14_S
ALN8_R
ALN11_S
ALN15_R
ALN6_S
ALN9_S
ALN19_S
